# Efficient and Consumer-Centered Item Detection and Classification with a Multicamera Network at High Ranges

**DOI:** 10.3390/s21144818

**Published:** 2021-07-14

**Authors:** Nils Mandischer, Tobias Huhn, Mathias Hüsing, Burkhard Corves

**Affiliations:** Machine Dynamics and Robotics (IGMR), Institute of Mechanism Theory, RWTH Aachen University, 52074 Aachen, Germany; huhn@igmr.rwth-aachen.de (T.H.); huesing@igmr.rwth-aachen.de (M.H.); corves@igmr.rwth-aachen.de (B.C.)

**Keywords:** computer vision, machine learning, unsupervised learning, segmentation, classification, camera network, workspace cognition

## Abstract

In the EU project SHAREWORK, methods are developed that allow humans and robots to collaborate in an industrial environment. One of the major contributions is a framework for task planning coupled with automated item detection and localization. In this work, we present the methods used for detecting and classifying items on the shop floor. Important in the context of SHAREWORK is the user-friendliness of the methodology. Thus, we renounce heavy-learning-based methods in favor of unsupervised segmentation coupled with lenient machine learning methods for classification. Our algorithm is a combination of established methods adjusted for fast and reliable item detection at high ranges of up to eight meters. In this work, we present the full pipeline from calibration, over segmentation to item classification in the industrial context. The pipeline is validated on a shop floor of 40 sqm and with up to nine different items and assemblies, reaching a mean accuracy of 84% at 0.85 Hz.

## 1. Introduction

In the European project “Safe and Effective Human–Robot Cooperation Towards a better Competitiveness on Current Automation Lack Manufacturing Processes” (SHAREWORK) robots are enabled to autonomously interact with human workers in low-automation industries. The objective is to develop new technologies and methodologies in different disciplines and combine them into a single framework that can be adapted to the needs of the specific use case. In this context, SHAREWORK brings together partners from research, public and national offices, service providers, and the manufacturing industry to fulfill this task. For autonomous interaction, the robots need to be enabled to sense and analyze their environment. The key technologies in this aspect are task planning, task identification, and environment cognition. First, the environment is analyzed to find the position of items and humans in the monitored space. From their spatial relations and interactions, tasks and objectives are derived. These are then provided to a knowledge base that is the base to automated task planning for a combination of stationary and mobile manipulators.

In industrial applications, 3D camera systems are used to perform varying checks on items and their geometry. 3D object recognition, however, is more common when it comes to the motion behavior of humans, e.g., in home safety [1]. This aspect of computer vision becomes more prominent in the verge of Industry 4.0, where machines are taught to interact in interconnected facilities. Even though the partial methods have been developed and deployed for many years, there is no comprehensive industrial application for 3D object recognition in wide workshop areas that is (a) easy to deploy, (b) fast, and (c) of acceptable accuracy. Our motivation is to design an item recognition system with multicamera usage that is fast in processing, easy to adapt to a multitude of use cases, and simple to use for untrained personnel. By this, our developments establish a basis to high-level robotic and automation tasks of the SHAREWORK framework (e.g., see [2]). This article displays the full work performed to achieve the objective, including network design, best practices and utility functionality, besides the main methodology that realizes the item recognition task.

**Figure 1:**
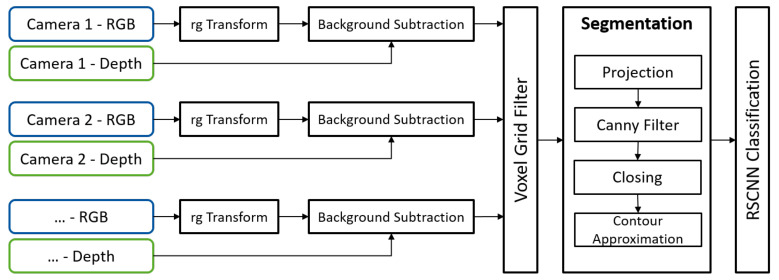
Methods used and data flow in the proposed pipeline.

To give an overview of the many methods used, Figure 1 depicts the data flow in the proposed pipeline. The structure of this article is organized as follows. First, the related work is presented in Section 2 covering topics from machine learning and classic segmentation. Furthermore, their industrial and consumer applications are discussed. In Section 3, we lay the design of the proposed end-to-end item recognition framework and hardware selection out. Next, the main methods are discussed in Section 4 (Segmentation) and Section 5 (Classification). Section 6 discusses the validation of the proposed methods in a lab scenario and gives best practices for item recognition system design. Finally, the content of this article is summarized and an outlook is given in Section 7.

## 2. Related Work

Ever since the launch of the Microsoft Kinect in late 2010, three-dimensional sensing technologies became much more affordable and available. Since then, usage of 3D sensors has surged, especially in recent years in the wake of autonomous driving, where LiDAR, radar, and camera systems are employed extensively. The raw sensor output of these sensors is in the format of a so-called point cloud. Item recognition is commonly based on 2D and 3D vision sensors and sensor networks. However, the methods developed in research and the ones used in the industrial practice differ heavily. While research is focused on complex machine learning, industry deploys more simple and classical methods. In order to improve the industrial standard, we combine methods from both domains to bring research to industrial use cases.

In this section, the related work in computer image analysis and industrial vision systems is introduced. Section 2.1 lists consumer and industrial object recognition systems and their commonly utilized segmentation methods. Afterward, Section 2.2 discusses common methods for item classification based on machine and deep learning techniques.

### 2.1. Item Detection in Practice

In industrial practice, most vision systems are designed to either detect and track shapes or to evaluate item quality based on their geometrical properties. Table 1 shows a selection of vision systems available that perform segmentation and classification tasks. From these vision systems, only one can perform classification out-of-the-box. However, most industrial vision tasks are designed in such a way that the item is known before it is analyzed by the vision sensor, e.g., by only inserting items of the same type. In this case, the vision sensor system only performs quality checks. However, the classification problem becomes more prominent in bin picking applications, where certain items need to be segmented and classified in a heap (e.g., [3,4]). In addition, vision systems are restricted in resolution and range. Thus, they are commonly operated only at short range or, if operated at longer ranges, only used to detect large items (e.g., ifm O3D). The only system performing a classification task is the HemiStereo DK1, which detects and counts people in a predefined area, used in stores. Thus, also in this case, the detected objects are large and only of two classes (i.e., human or nonhuman). Multiclass detection is currently not part of industrial applications.

As emphasized by Table 1, parts segmentation already has an important role in the manufacturing industry. According to Shleymovich, Madvedev, and Lyasheva [5], those methods are usually based on four different types of approaches: contour detection, morphological operations, threshold segmentation, or clustering.

**Contour detection** is the task to find the outlines of an object. The major challenge is the edge feature detection. The most common methods for this are Canny edge detection and blob detection. The blob detector is based on the Laplacian of Gaussians as introduced by Lindeberg [6]. Blobs are chains of pixels that share similar features, e.g., color intensities. Based on the Laplacian blobs, Lowe [7] proposed the scale-invariant feature transform (SIFT) that is also used for end-to-end classification, mapping, and other robotics-related tasks. On the other hand, Canny [8] introduced the Canny edge detector that finds contours based on gradients (see Section 4.3.2). Another type of method is **mathematical morphology**, which finds structures based on geometric features and topology [9]. **Thresholding** extracts binary images from grayscale by setting threshold values. Depending on the method used, the thresholds can be fixed, dynamic, or multilayered (e.g., see [10]). **Clustering** is a wide field of methods utilized to group similar data points based on specific metrics. These methods range from simple, e.g., Euclidean clustering, to complex, e.g., fuzzy clustering by local approximation of memberships (FLAME) [11]. The major benefit of the discussed methods is that they are comparatively fast to implement and validate, even for personnel less trained in machine vision. However, the four types of classic segmentation are more and more replaced in research by machine learning approaches, given a satisfactory amount of training data and knowledge.

### 2.2. Computer Vision and Learning Techniques

To recognize the content inside a point cloud or a point cloud segment, classification is performed. As a closed algorithm solution is next to impossible, neural networks are applied to this task currently. Although methods to work on voxel structures are available, the most successful approaches in point cloud classification according to the ModelNet leaderboard (https://modelnet.cs.princeton.edu/; accessed: 14 July 2021) are able to directly process 3D point cloud data. The PointNet neural network architecture, proposed by Qi et al. [12], pioneered in this category. While PointNet only processed each point separately, its successor PointNet++ [13] takes spatial relations between points into consideration, similar to convolutions in image processing. This allows the distinction of local features, of which again abstract, higher level features can be derived. Encoding more information, the dynamic graph convolutional neural network (DGCNN) architecture, proposed by Wang et al. [14], adds the distance in feature space to the *k* closest points explicitly to the data presented to the neural network. The architecture also allowed for faster computation, needing only a seventh of the time for the forward pass compared to PointNet++. Among other changes, the addition of the absolute distance to the representation of points in proximity leads to the relation-shape convolutional neural network (RSCNN) proposed by Liu et al. [15]. Here, the closest points are determined by ball query, instead of *k*-nearest neighbors (kNN).

Most of the networks for use in point cloud classification can be extended to perform segmentation as well (called end-to-end detection). This is generally performed by reversing the abstraction layers to form deconvolutional layers (see e.g., [13,14] or [15]). However, this would require the availability of sufficient training data. For data generation, however, the operator would be required to label each point of each point cloud manually. This would contradict with the concept of intuitive operation and usage for untrained personnel.

## 3. System Design

Before the methodology is discussed in Section 4 and Section 5, this section provides an overview over the hardware and software design. All methods are implemented with the Robot Operating System (ROS). The SHAREWORK framework aims to offer lenient methods for computer vision. The facility personnel that deals with the technology cannot be bothered with collecting training data or annotating data sets. Furthermore, the methods need to be adjustable to different use cases and facilities. In SHAREWORK, four different use cases (https://sharework-project.eu/; accessed: 14 July 2021) from diverse industrial sectors are addressed. Thus, our aim is to reach some—in the community unusual—specifications, that allow to ease usage of the system:

- Use as few data as possible
- Ease data annotation
- Allow high ranges and a multiplicity of item geometries
- Maintain fast cycle times for further processing in high-level tasks

### 3.1. Hardware Selection

To allow detection of small items at high ranges, only 3D cameras with extraordinary high resolution can be used. In a market research, it soon became obvious that the highest resolution consumer camera (to this date) is the Stereolabs ZED with 4 MP. To our knowledge, there is no industrial 3D camera with higher resolution that can be used in the presence of humans (see Table 1). However, using such high-resolution 3D sensors at potentially high frequencies (here: 10 Hz) requires specific interfaces. Therefore, the four cameras are individually connected via fiber optics to the central processing server. To power the system, the server has to have high-end computational capability. We use a system with two Nvidia Quadro RTX6000 GPUs, two Intel Xeon Gold 6130 CPUs, and 192 GB of RAM. This system is later used in validation (see Section 6). With this setup, one scan can have up to 10,969,344 data points, which ultimately results in a data load of approximately 3 GB/sec at a frequency of 10 Hz.

### 3.2. System and Software Architecture

To cope with the hard requirements and the high data load, the system architecture is designed as a slim process. We will later show that the pipeline can be computed in approximately 1200 ms in Section 6.1. For further processing, 3D segments of the desired items are required. However, using native 3D methods on such high data load is impossible even with a high-end setup. Therefore, our objective is to use 3D vision where necessary and 2D vision or projections whenever possible. In addition, some processes are outsourced into offline steps that need to be applied prior to the recognition application (e.g., background initialization; see Section 4.2). This results in the specifications stated in Table 2. Certain processes need to be synchronized due to two reasons:

1. As point clouds are generated from stereo vision, they always arrive after the 2D image (left lens) and need to be synchronized before processing
2. After processing, either point cloud or image, and if afterward, the other type should be included, the processed result and other data type need to be synchronized

Without synchronization, it is not guaranteed that corresponding data are processed, which may result in misalignment or malformed projections. An approximate time scheme is used to cope with this challenge. However, a delay is induced between the first arrival of data and the last corresponding input. In the approximate time scheme, all arriving data are held back in queues. Once all queues have data, the last arrival is named the pivotal point. From this data set, time deltas are calculated toward sets in the other queues. When all candidates are chosen, the now synchronized data sets are forwarded.

### 3.3. Network Calibration

The cameras are calibrated toward a single global coordinate frame. We use a ChAruco board as calibration target, i.e., a chessboard pattern with embedded AR tags on the white elements. The cameras span an area of 40 sqm (8×5 m). With a resolution of only 4 MP (2208 × 1242 px), calibration at this range is challenging. The method behind our calibration algorithm is a point-to-point association. Within, the corners of the AR tags on the ChAruco board are detected and identified (AR tags have a unique ID). Additionally, the chessboard edges are used for higher robustness. Based on the known dimensions of the calibration target, the detected features are compared with the expected features. This again is based on the known projection behavior from the 3D environment onto the 2D image plane of the camera (pinhole camera model). If a satisfactory amount of point-to-point associations can be established, the position and orientation of the calibration target relative to the camera can be estimated. Finally, transforming all cameras to the target coordinate system gives the relative poses of the cameras. While the approach is well established for camera calibration (e.g., Lepetit, Moreno-Noguer, and Fua [16]), it requires the cameras to detect the individual black square elements of the AR tags. In our case, the tags have 4 × 4 elements. When the target is located in the middle of the monitored space, some sensors are unable to detect the tags even though the target board is printed on DIN-A0 (tag size >20 cm).

**Figure 2:**
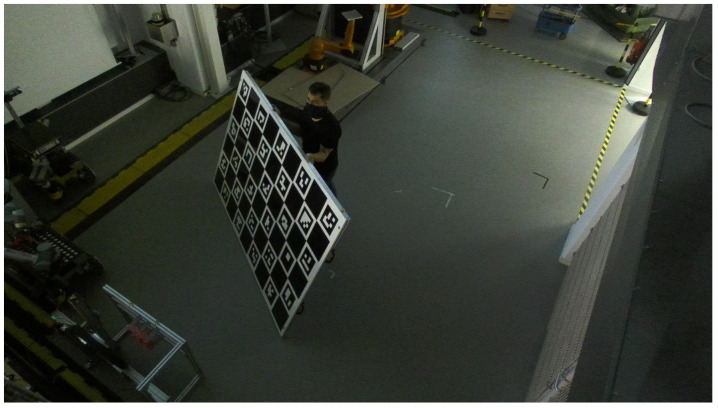
Cumbersome calibration board used to achieve high accuracy calibration.

**Figure 3:**
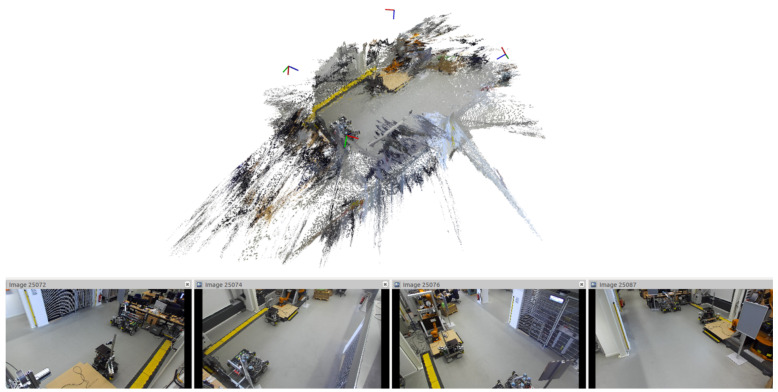
Calibrated point cloud of IGMR lab at 0.2 px cumulative calibration error; coordinate systems indicate camera poses.

There are two options to counteract this challenge: either print a bigger board or establish more relative transforms. Naturally, a bigger board directly solves the issue. We printed the target on 2.23×1.5 m (1.5 m is the maximum width for most industrial plotters). With this board, we can achieve a cumulative calibration error of 0.15 px (see Figure 2). While the error is good, the board is cumbersome and needs to be operated (mostly) by several people. Thus, a second option is to find positions in which multiple cameras can detect the smaller board. If the same camera is part of two different position sets, the relative poses between all involved cameras can be established. For this approach, first, one camera is calibrated precisely toward the global target. Afterward, pairs are made and transforms are established. In our case, this approach realizes a cumulative calibration error of 0.4 px (see Figure 3). The latter approach is the more practical approach, but it is subject to the particular items whether the precision realized is sufficient. It is to be noted that pixels become large at a range of up to 10 m. In the validation (Section 6), we use the less accurate approach.

## 4. Unsupervised Segmentation

The first major operation is to segment the point cloud into objects of interest. As annotating a load of teaching data is not suitable for untrained personnel, we aim to design the process as unsupervised segmentation. To achieve this objective, while also maintaining fast cycle times, we have to use slim but powerful methods. By these measures, the proposed pipeline is generic and can be easily adjusted to any use case.

In this section, first, the methodology and pipeline are discussed (Section 4.1). Afterward, the main methods are presented (Section 4.2 and Section 4.3). Finally, the benefits and drawbacks of the methodology are discussed (Section 4.4).

### 4.1. Methodology

To allow fast cycle times, the first problem is to reduce the data load and to structure the leftover data for fast access. In general, 2D methods are much faster than 3D methods. Thus, we design the system to use 2D image processing to reduce the 3D data set before computing native methods on the point cloud. This requires intensive prefiltering.

**Figure 4:**
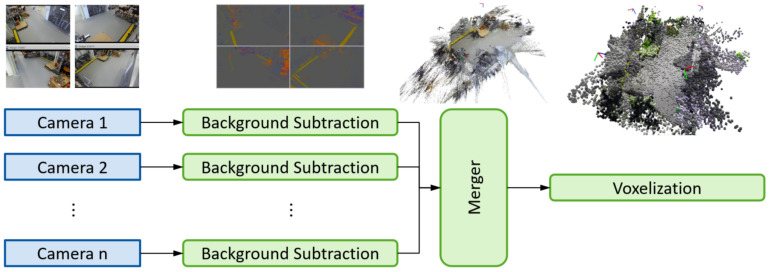
Prefilter pipeline from raw camera input to merged and voxel-abstracted point cloud.

The full prefiltering pipeline is depicted in Figure 4. The system is designed for an arbitrary number of input devices. The only limiting factor on the number of cameras is the computational hardware (in our case, one GPU per two cameras). First, for every camera, a 2D background image is generated offline. These background models are then used to cut static objects from the incoming images. The leftover dynamic objects are merged and stored in a voxel grid data structure. Even though merger and voxel grid appear as two separate steps in Figure 4, they are combined to reduce redundant operations. All prefiltering steps are parallelized for minimal cycle times.

To establish object segments, the voxel grid is first projected into a 2D image. On this projected image, contour detection is performed to separate the target items. Finally, the contours are projected onto the original point cloud to get the 3D segments.

### 4.2. Prefiltering with Background Subtraction

The process of generating a mean background model and distinguishing between foreground and background objects is called background subtraction. In this article, background subtraction is used to reduce the problem size while analyzing the point cloud for segments. As stated in Section 3.2, this method requires synchronization and may induce lag into the system. We first perform background subtraction on the 2D images inherently generated by the 3D cameras and then cut the dynamic objects from the point cloud for further processing.

#### 4.2.1. Color Space Transform

One major drawback of operations performed on the native RGB color space is that shadows induced by the environment appear as different color codes. Hence, comparing objects partially occluded by shadows or in different lighting scenarios is hard. The RGB color space is defined by the three components $i_{RGB}={\left[R,G,B\right]}^{T}$, where $i_{RGB}$ is the color intensity vector. The color components *R*, *G*, and *B* are integer values in the range of 0 to 255, where ${\left[0,0,0\right]}^{T}$ is black and ${\left[255,255,255\right]}^{T}$ is white. Black and white further correspond to absence or full presence of light. Similarly, different colors can be generated by mixing the component intensities.

By normalizing the color components, they are transformed in the rg chromaticity space $i_{rg}={\left[r,g\right]}^{T}$, given by

(1) $$\begin{array}{c} r=\frac{R}{R+G+B},\;g=\frac{G}{R+G+B},\;1=r+g+b. \end{array}$$

**Figure 5:**
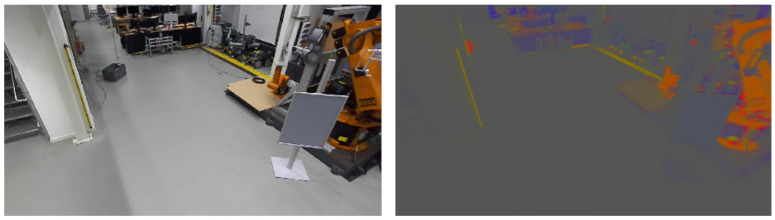
Comparison of RGB image (**left**) and rg chromaticity image (**right**) of the same scene.

In this color space, objects have the same color intensity vector in different lighting scenarios—given they have a homogeneous color. An example from our lab is depicted in Figure 5. Note that the shadows disappear in the right image. However, rg chromaticity has the drawback that objects also disappear, which have the same color as the background they are placed on. Thus, it is advised to either use RGB with a well lit work place or rg chromaticity with a work place of different color than the desired tools and items. For validation (Section 6), we use rg chromaticity.

#### 4.2.2. Background Subtraction

After the color transform, background subtraction is applied. The method is split into two stages. First, a mean background model is generated. Afterward, incoming images are filtered to get a foreground mask. The output of background subtraction is a binary mask, where foreground objects are denoted 1. The method is based on an adaptive mixture of Gaussians (MOG) model established by Zivkovic [17]. In contrast to the original method, we prevent the background from updating after converging to the original workspace. Thus, not only dynamic but also static objects brought into the monitored space will appear in the foreground mask. The foreground mask is then projected onto the point cloud, from which the foreground 3D objects are isolated. This process is run in parallel for all cameras connected to the network. A single background model is based on a history of approximately 1000 images and takes five to ten minutes to generate. As the background model is generated prior to the recognition process, the model will not incorporate static objects placed into the environment at a later point of time. We suggest to remove all movable objects from the environment beforehand. Thus, temporarily static objects will appear in the segmentation data but will be sorted out by the classification. In case of major layout changes, a new background model needs to be generated.

**Figure 6:**
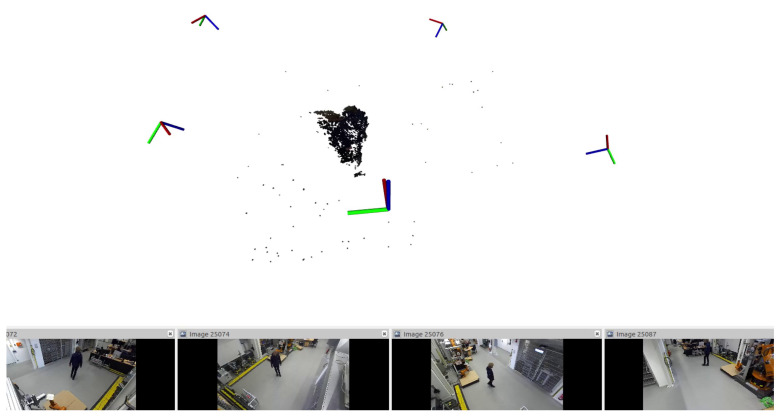
Foreground point cloud of a walking person after merging, generated by background subtraction on rg chromaticity color space.

We observe that some pixels tend to flicker, i.e., a continuous shift in color intensity. This effect is introduced by the rg chromaticity transform. To counteract this behavior, a flicker filter is trained in parallel to the background model. In the filter, each pixel is observed for a specific time duration. If its allocation changes from background to foreground (or vice versa) in this period, the probability for flicker is increased. If it does not change, the probability is lowered. We use an observation period of five consecutive frames. Furthermore, the start of training is delayed, because in the first frames, the background model is subject to many changes. These frames cause the flicker filter to diverge. Once trained, a pixel indicated as flicker is prevented from being added to the foreground mask, hence it is always treated as background. In practice, this filter lowers the overall noise in the background subtraction with prior rg chromaticity transform. The resulting foreground point cloud is depicted in Figure 6.

### 4.3. Contour Detection

**Figure 7:**
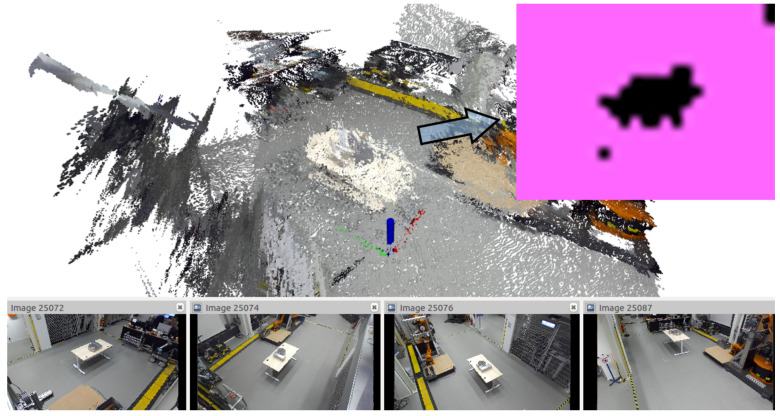
Projection of the foreground point cloud onto a ground-parallel image plane (pink background image). The figure depicts the full merged point cloud. The ROI is set to 2×2.5 m centered around the table. Foreground pixels are colored black.

Once the foreground point clouds have been isolated, they are merged and put into a voxel grid (Section 4.3.1). Afterward, the voxel are projected onto an image parallel to the ground plane. Colors are discarded. The projection is depicted in Figure 7. The projection should be restricted to few regions of interest (ROIs) to save computation ressources. Typical ROIs are work benches or tool racks. On the projected image, contours are found by applying a combination of chain approximation and morphologic operations (Section 4.3.2). Afterward, the contours are projected back onto the foreground point cloud to separate items into segments.

#### 4.3.1. Voxel Filter

The voxel filter is used to unify the point cloud and reduce noise. A voxel grid is a structure in which larger cubes are split into smaller cubes when a data point is added to the structure. This typically results in regions of different discretization. However, as we utilize the voxel grid for projection, the grid is initially generated with a fixed depth and according number of points. Thus, it is possible to specify the side length of each voxel. The side length is chosen so that the mean depth perception error of the cameras vanishes, i.e., 0.1 m in our case. By this, additional filters can be waived. However, outliers will still be part of the voxel grid. Another benefit of the voxel grid with fixed size is that the structure can be initialized offline and then filled online in a parallelized fashion. Hence, the process of abstracting the point cloud into voxels (“voxelization”) is fast.

The voxel grid is oriented according to the global coordinate system. Parallel to its *x*-*y*-plane (cf. Figure 6 red and green axes), the grid is projected in the *z* direction (blue axis). The projection height can be specified, e.g., to find objects covered by a robot. As only outer contours should be detected later on, colors can be discarded to save memory and spare a transform in the edge filter (see Section 4.3.2). To improve edge and contour detection, the projected image’s pixel count is increased by splitting every individual pixel into multiple subpixels (here: 16). This prevents small contours from merging into one larger contour.

#### 4.3.2. Contour Approximation

**Figure 8:**
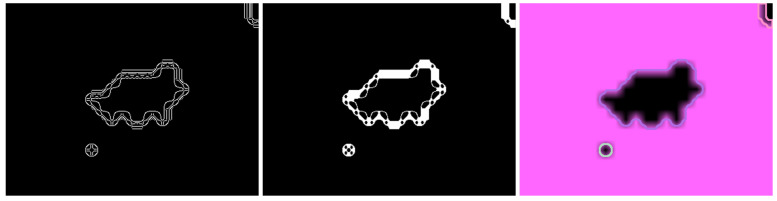
Contour detection pipeline; from left: Canny edge detection, morphologic closing, chain approximation.

To isolate the segmented objects, the projected image is further processed. The pipeline is depicted in Figure 8. First, Canny edge detection [8] is applied to detect the edges in the image. The Canny edge detection algorithm first reduces noise by applying a Gaussian filter. Then, the magnitude and orientation of image gradients is computed. With the gradients known, edges are compared and thinned depending on the edge strength. This is done with nonmaximum suppression using a moving window filter. Afterward, the edges are classified into strong and weak edges using a double threshold (here, 40 and 80). Afterward, a hysteresis filter is used to argue on the weak edges. The filter is used to track and keep edges with at least one strong edge. The edges detected are precise, but they tend to generate open contour chains.

To close open contour chains, morphologic closing (see Dougherty [18]) is applied. Morphologic operations define a kernel (here, ellipsis of kernel size two) in which 1-pixels are counted in the binary image. Closing consists of first erosion and then dilation. In erosion, if all pixels are one, the output pixel is set to one, and zero otherwise. In dilation, if at least one pixel is one, the output pixel is set to one. Consequently, erosions thins the edges, while dilation inflates them, hence morphologic closing results in filled shapes.

After closing, topological chain approximation according to Suzuki and Abe [19] is applied to find the final contours. The algorithm determines a contour starting point from which it follows a contour. If another contour is intersecting or inlaying, it is ordered hierarchically in a tree structure accordingly. Hence, the algorithm establishes a contour hierarchy, which can be used for postprocessing. The main advantage over similar algorithms is the low computational complexity. After the contours have been found, their enclosed area is computed and small contours are discarded. The contour is then projected onto the merged point cloud to generate segments. Again, small segments (here, less than 50 points) are discarded, because they tend to result in malformed classifications (see Section 5). The resulting segments correspond to the items in the monitored ROI.

### 4.4. Discussion of the Proposed Method

As will be shown later (Section 6), the proposed method can fulfill all demands stated in Section 3. In particular, the segmentation method reaches fast cycle times and high accuracy measures for low item counts. Furthermore, the handling for untrained personnel is easy, as the only requirement is to capture a background model beforehand, which requires low effort. However, due to this design decisions, some drawbacks have been introduced. On one hand, items have to be separated by at least one empty voxel to be distinguishable—in this case, 10 cm. On the other hand, objects with spatial relations along the global *z*-axis (e.g., stacks or overlap) are not separated. Latter can be solved with the proposed method by not converting the projected image into binary but keeping the color gradients and, therefore, detecting more inlaying edges. The chain approximation will directly establish the semantic structure (i.e., contour hierarchy) and indicate which object is stacked or inserted into which other object. As in SHAREWORK, those spatial relations are not a concern for the moment, and thus it can be assumed that this is the case for most industrial use cases with simple manufacturing tasks (e.g., screwing, drilling, or applying adhesives), the spatial relations have been discarded for overall detection speed.

## 5. Classification

After segments have been established by the algorithm proposed in the former section, the next step is to identify which items are included in the segmented point clouds. Therefore, a convolutional neural network (CNN) classifier is used. This section elaborates on the methods used. First, the training data are discussed in Section 5.1. Afterward, the CNN used and the classifier methodology are discussed in Section 5.2.

### 5.1. Training Data Generation

When we started with the project, the classification networks were trained on artificial data from data libraries, CAD, or similar sources. However, due to the widely different characteristics of the camera network used in our use case, the networks failed to generalize on real data. This issue is often left out of consideration once it has been shown that a specific architecture reaches a good accuracy measure on artificial data. However, when transporting machine learning into practice, it is inherently important that the networks can deal with real data. Thus, we decided to train the network on data generated with the segmentation method proposed in Section 4. This data collection approach has two benefits. On one hand, it is easy for untrained personnel to generate new data sets by just putting the desired items into the workspace. On the other hand, the real items and environmental influences can be captured in the data set. In addition, the more the system is brought into different applications, the more data are generated and collected, and, therefore, more items can be detected in different use cases, given that data collection is not restricted by the use case owner.

**Figure 9:**
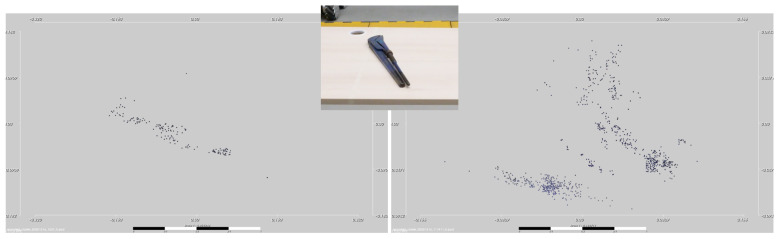
Two data samples in the training set “tongs” taken from different poses in the environment.

However, when capturing data with a network of stereo vision cameras, the item point clouds become dependent on the item’s orientation and position toward each individual camera. This is emphasized by Figure 9. While some items have distinct geometrical contours, others are washed out. The latter case (depicted on the right side) appears when broad but flat object sides face the camera in a flat angle (compare Figure 7 to the most-right camera image). By this, even on the small surface area of a table, there is a vast difference in how items appear in the point cloud. To generate a good data set, the operator is required to work conscientiously and cover most of the desired workspace to compensate potential differences in the object shape. However, by generating the data directly with the real camera network and the segmentation used in practice, the classification network is enabled to learn and generalize on real data and, therefore, reaches high accuracy even with nonoptimal data sets.

### 5.2. Item Classification with Neural Networks

With a variety of different neural network architectures for point cloud classification available, we evaluate on which to use in the described task. In literature, most architectures are benchmarked and compared on data from the ModelNet data set which comprises of artificial data. However, information on the performance on real data is rarely available, especially as the exact distribution of noise varies depending on the type of camera used. Furthermore, no information on handling multiple 3D perspectives of the same object is available. For this reason, we design a data set for the comparison of architectures, which is similar to the data sets expected as user input in the later applications. Each sample in this set consists of two point clouds of the same object from different camera perspectives. The data set is designed in a way such that classes can easily be discerned by a human but contain similar shapes and thus challenge the neural network. Note that this set is not to be confused with the set proposed in Section 6. It consists of the following six classes:

- box: Various cuboid boxes with different printing patterns on their surfaces
- coiledcable: Cables of different lengths and colors in a coil, leading to point clouds with a variety of densities and irregular shapes
- cup: Cups of different shapes, colors, and prints
- filamentroll: Rolls of 3D printing filament with different colors of filament and spools
- pliers: Pliers of different types, e.g., clipping pliers, engineer’s pliers, crimping pliers
- taperoll: Tape of various types on rolls

The data set is split up in two ways to test for different abilities of the networks separately. The first split is used to prove the ability of the neural network to recognize known objects. For this, point clouds of the same object from different angles are placed in the training as well as the evaluation set. The second split gives a rough understanding of the neural networks ability to generalize; no point cloud of an object in the training set can be found in the evaluation set and vice versa. To achieve higher scores, the neural networks have to abstract on general features of classes.

**Figure 10:**
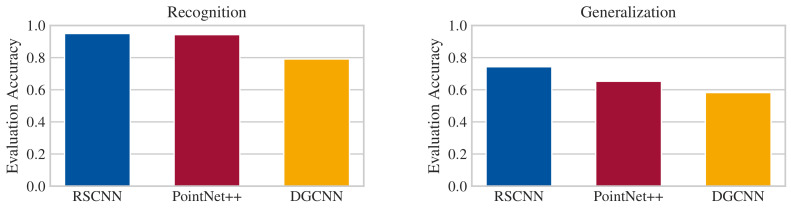
Evaluation accuracies of different neural network architectures with respect to the split of the data set.

For neural network architectures, PointNet++, DGCNN, and relation-shape CNN are evaluated. At the time of writing, relation-shape CNN achieved the best results in point cloud classification on point clouds according to the ModelNet leaderboard. Architectures providing better results do not consume point clouds directly but rely on images created from multiple view points. This approach is not applicable for our use case, and thus is disregarded. The RSCNN network architecture uses the approaches of PointNet(++), and applies improvement strategies similar to DGCNN. With the difference in results on the ModelNet benchmark being only a few percentage points, PointNet++ and DGCNN are also taken into consideration for validation on application data. They are trained with an Adam optimizer with a learning rate of 0.01 and 2048 input points. Gaussian zero-mean noise with a standard deviation of 0.005 is added to all input channels for augmentation. In the evaluation, RSCNN outperforms both PointNet++ and DGCNN in terms of accuracy in recognition as well as generalization ability, which is also shown in Figure 10. Additionally, RSCNN is less restrictive regarding its memory usage in comparison to PointNet++. On our data set, RSCNN scores 95% accuracy and is able to produce the classification result in about 250 ms. Furthermore, it proves resilient if trained with a smaller number of points; even with only 128 points per point cloud, only negligible differences in accuracy are observed. The forward time for this amount of points reduces to approximately 100 ms. These different factors lead us to select RSCNN as classification network.

**Figure 11:**
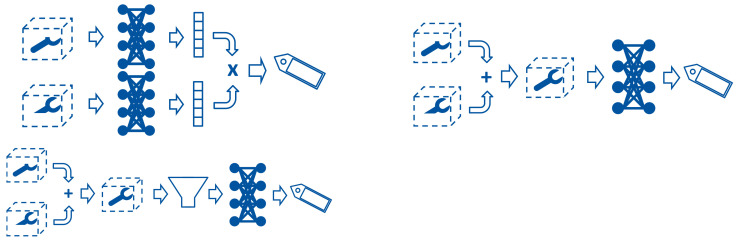
Strategies evaluated for handling of multiple point clouds with similar content (separate classification, concatenation, concatenation, and filtering).

Furthermore, to handle the different camera perspectives, three strategies are compared, which are illustrated in Figure 11:

1. Classifying each point cloud separately, and fusing the probabilities
2. Concatenating the point information in global coordinates before classifying
3. Concatenating the point information in global coordinates and applying a voxel filter subsequently to reduce redundant data before classifying

The evaluation of the three strategies does not show a superiority of one over the others, as all show the same level of performance and increasing complexity. As expected, adding a perspective generally increases the classification accuracy by approximately 2%. Due to this, we choose to provide the neural network with a voxel-filtered point cloud, as this is data already available in our pipeline. Compared to considering point clouds separately, it has the advantage that classification only has to be performed once, allowing for a better prediction of time needed and less additional parallelization required.

## 6. Validation

This section presents the validation of the proposed pipeline and methodology. As the accuracy of the classification is directly dependent on the quality of the unsupervised segmentation, it is reasonable to test both modules separately on the same data set. In the following, we describe the test setup (Section 6.1) and the results of segmentation (Section 6.2) and classification (Section 6.3).

### 6.1. Test Setup

To bring the scenario closer to real-world applications, a table is set up in the middle of the monitored space. On the table, a total of nine items are placed in different configurations. To train the neural network, the items are placed, rotated, and moved individually on the table. By this, a data set of 3730 segmented objects is generated.

**Figure 12:**
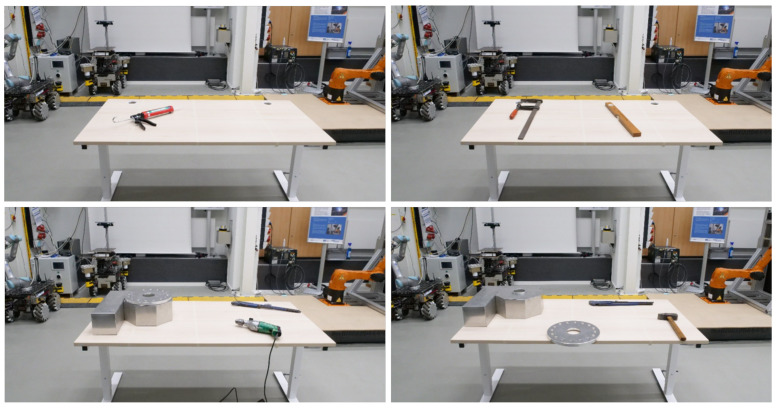
Exemplary four scenarios chosen to depict all items and the table setup (1 item: silicone gun; 2 items: clamp, bar; 3 items: rotary table (full), drill, tongs; 4 items: rotary table (LA), rotary disk, tongs, hammer).

For validation, 45 scenarios are defined. Each scenario is recorded for 2 minutes. As the camera publishing frequency in the ROS network is not constant, there is no exact frame count, but the upper limit is 1200 frames (10 Hz) per camera and scenario. A typical manufacturing task in SHAREWORK features one to two items. Eighteen scenarios feature single items (two of each class) and ten feature two items. As the discretization chosen requires items to be placed with a distance of 10 cm and the table being 1.6 × 0.8 m, the limit of objects that can be placed is four. Hence, the other scenarios have three (11 cases) and four (6 cases) items. Note that no SHAREWORK use case requires four items to be detected simultaneously. The items have a varying count of appearances, as some are too large to feature in some scenarios. The counts are listed in Table 3. Rotary disk and rotary table (LA; short for “Lower Assembly”) can be combined into the class “rotary table (full)”. This is a model of a ZPGI–Servo rotary table by Spanish manufacturer Goizper (https://www.goizper.com/en/industrial/index-units-servo-rotary-tables/zpgi-servo-rotary-tables; accessed: 14 July 2021). Exemplary items and some scenarios are depicted in Figure 12.

The hardware used for validation has already been stated in Section 3.1. Throughout the tests, the system reaches a mean cycle time of 1176 ms or approximately 0.85 Hz. Given the data load and complexity of the task, this satisfies the agility conditions. For robotic task planning and human task identification, a cycle time of 2000 ms was stated as tolerable by the SHAREWORK developers; hence, this requirement is fulfilled.

### 6.2. Validation of Segmentation

The segmentation is tested with a confusion matrix, i.e., the scores true positives (TP; items present and correctly segmented), true negatives (TN; items not present and not segmented), false positives (FP; items not present, but segmented), and false negatives (FN; items present, but not segmented). As the proposed method does not reject false segments, the TN score is not possible to be applied. In total, 9652 items are sampled. The confusion matrix is listed in Table 4.

**Figure 13:**
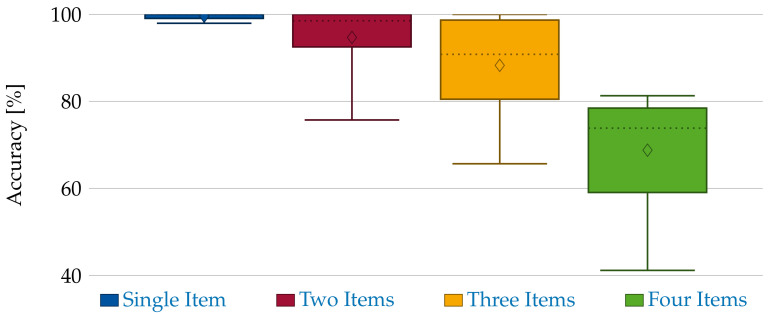
Accuracy measures and variances depending on the number of items in the scenario.

This results in an accuracy score of 87.84% (TP + TN over all samples). Furthermore, the method tends to rather not depict present items than generating wrong segments. This is based on the discretization used to shrink the problem size. Hence, close objects tend to be merged into a single object, which results in FN. While this establishes a mean performance for the segmentation, it is also of interest how the method performs depending on the number of items in the workspace. Therefore, Figure 13 shows the accuracy scores for each scenario. This shows that the mean accuracy is mainly influenced by the scenarios with higher item count. For less items, higher accuracies can be achieved. The mean accuracy in the scenarios with one or two items is 99.58% and 94.7%, respectively. However, while this method is well suited for less or widespread items, it fails when many items are put into a small space, e.g., when applied to bin picking.

**Figure 14:**
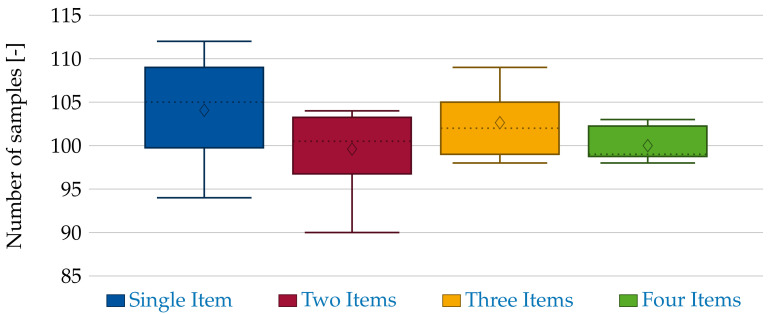
Number of segmented objects per two minutes and scenario.

In addition to the accuracy, we test the influence of the item count onto the mean cycle time. The results are depicted in Figure 14. The mean output sample size of all scenarios is between 95 and 105 samples all tending toward 101–102 frames. Hence, these results indicate that the method is not dependent on the number of items in the workspace.

### 6.3. Validation of Classification

**Figure 15:**
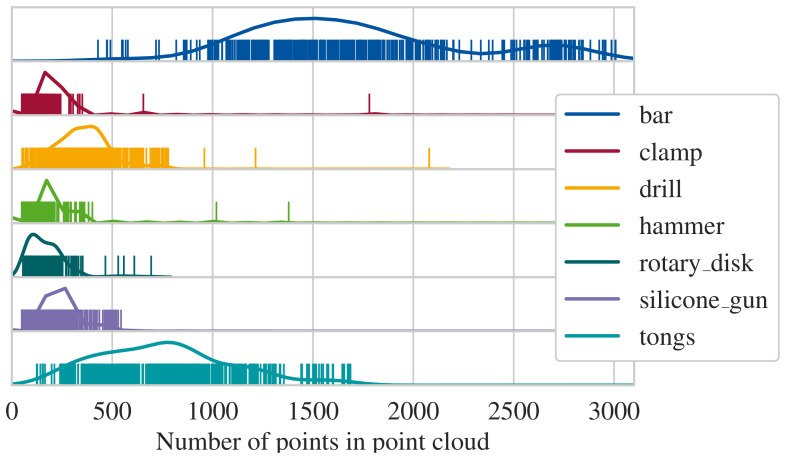
Distribution of points per point cloud; vertical lines symbolize a single sample.

**Figure 16:**
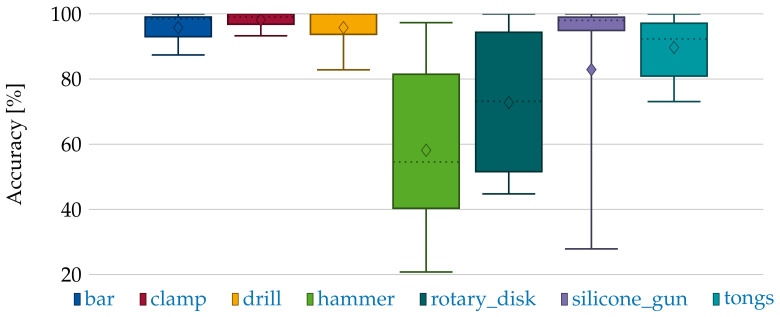
Accuracy measures for the classified items without rotary table full and LA.

For the classification, the segments generated in the last section are forwarded to the classifier module. We observe that the classifier is unable to differentiate between the two rotary table classes (LA and full). Thus, they are removed from the testing data, resulting in a data set with seven items shown in Figure 15. As apparent, the number of points per point cloud are less than expected compared to Section 5.2. For this reason, the number of points input into the neural network is reduced to 128, as a replication of points results in a more drastic loss of information than downsampling. This is due to the structure of the selected neural network. For the comprehension of the surroundings, points in a specific distance to selected points are queried. However, the number of points in this query has an upper limit. If the queried point has been replicated to fit to the number of input points for the neural network, the surrounding points are congested with the replications, leaving next to no space for points which would contribute information. The accuracy values after this adaption are shown in Figure 16. The mean accuracy is 84%. It should be noted that the results are relative to the accuracy of the segmentation because FP and FN are also forwarded to classification. We did not filter the segmented objects, as the real-world application does not allow to manually filter out malformed segments. The dependency is prominent in the classification of the silicone gun class. Here, most segments are classified properly, but a single case fails to establish proper segments and, therefore, impacts the score. Hence, the mean accuracy of 84% can also be understood as the mean accuracy of the system end-to-end. However, for most classes, the accuracy is even higher. For the smallest item (hammer), the error variance is high. This is because the segmented point clouds of the hammer has much fewer points than the other items. We observe that a greater number of points in a point cloud is better for classification. For the items selected, the point cloud size deviates by multiple orders. In particular, depending on the orientation toward the cameras, the point count for one item can deviate between less than ten and multiple hundred points. Thus, we set a minimum of 100 points as a reasonable lower bound for the classification.

The classifier itself has comparatively no significant impact on the computational time. The main contributors to the cycle time are the operations performed on the full point cloud, i.e., background subtraction and voxel grid.

### 6.4. Discussion of Results

The proposed work underlines some aspects of machine learning in application. There needs to be a balance between range, accuracy, detectable object size, and run time. As we tried to optimize all of these aspects, other trade-offs had to be made. On the beneficial side, the system runs at almost 1 Hz with reasonable accuracy and while monitoring an area of 40 sqm. To our knowledge, such a setup has not been implemented before. On the downside, the minimum item size is directly impacted by the resolution of the cameras and the minimum point cloud size for classification of 100 points. The hammer (see Figure 12) is the smallest detectable item in the data set and also has the worst classification accuracy. Hence, there is a lower bound on item size. In addition, due to the characteristics of the proposed methodology, items cannot be stacked or overlapped; they must be separated by a single voxel side length (here 10 cm). Further, even though the process makes it easier for untrained personnel to deal with machine learning and computer vision, e.g., by the automated training data generation by segmentation, data are still needed for every item class. We further observe that stereo vision is less beneficial than other 3D measurement techniques, as it causes the items to appear widely different at varying positions and orientations. We assume that this will not be the case for time-of-flight cameras or 3D LiDAR as indicated by other SHAREWORK developers. Hence, with those technologies, the need to capture items at various locations in the workspace may be reduced.

For future setups, trade-offs are identified which will help interested people to design their object detection network. We hope that these lessons learned will provide some guidelines to such system design.

- **Workspace area vs. item size**
  The larger the area monitored, the larger the items need to be in order to be detectable.
- **Market availability vs. item size**
  Check whether cameras of desired resolution are available; otherwise, it will impact the minimum detectable size
- **Camera resolution vs. computational time vs. budget**
  The higher the camera resolution, the more points have to be processed. Thus, higher resolutions are more demanding on the prefiltering steps, and hence impact computational time. Furthermore, higher data load may require specialized interfaces and hardware, which impacts the budget.
- **Item knowledge vs. spatial relations detectable**
  To distinguish between objects that appear as a single entity (e.g., inserted objects), an unsupervised segmentation method is unsuited. To differentiate between more spatial relations, supervised learning methods should be used, which again make the system less user-friendly and demand more data.
- **Grade of discretization vs. computational time vs. item size**
  When a voxel filter is used the level of discretization directly correlates with the computational time. However, the larger the voxels, the larger the minimal detectable item size.

## 7. Conclusions and Outlook

In this article we presented an end-to-end approach for large area item detection and classification with a network of 3D cameras. The approach is based on a combination of unsupervised segmentation, efficient prefiltering, and neural network classification. By design, the method eases usage for untrained personnel and is adaptable to other scenarios and industries. To show this, we validated the approach with a novel data set generated with four Stereolabs ZED cameras at long ranges that consists of 3730 training data samples and 9652 segmented validation samples, besides 90 min of recorded raw 3D data. The proposed end-to-end detection and classification pipeline shows reliable results at short run times of 2 min. We discussed the method and show benefits and downsides induced by the hard requirements of SHAREWORK. Finally, we presented trade-offs that can be used for design of detection networks in future applications.

As a next step, the proposed method is integrated into the overall SHAREWORK framework. In the ongoing integration steps, full and partial methods are brought directly to our industrial partners. While our methodology is comprehensive as a combination of partial methods, these partial methods can still be improved. The background subtraction method could be adjusted to directly incorporate 3D data instead of 2D data and then projecting it onto the 3D point cloud. In addition, the contour detection should be tested against blob analysis and other contour detection methods such as, e.g., watershed transformation. In the field of machine learning, new innovations are made continuously, as this is a comparatively young field of research. We expect that new technologies will emerge, that can either fulfill the classification task more reliable with less data or directly perform end-to-end classification on their own without the need of elaborate annotated data sets.

## Figures and Tables

**Table 1:** Comparison of industrial item detection applications; range and rate refer to maximum values.

Manufacturer	Model	Resolution [px]	Range [m]	Rate [Hz]	3D Vision	Segmentation	Classification
ifm	O2D222	640 × 480	2	20	Yes	Yes	No
ifm	O3D	352 × 264	8	25	Yes	Yes	No
SICK	Visionary-B	n.a.	72	5	Yes	Yes	No
Stereolabs	ZED-2	2208 × 1242	20	15	Yes	Yes ${}^{a}$	No
Orbbec	Astra Pro	1280 × 720	8	30	Yes	Yes ${}^{a}$	No
ams	BELICE	5900	n.a.	n.a.	Yes	Yes	No
Vistion	IN-SIGHT	800 × 600	-	55	No	Yes	No
Intel	Realsense LiDAR	1920 × 1080	9	10	Yes	Yes ${}^{b}$	No
HemiStereo	DK1	1200 × 1200	5	n.a.	Yes	Yes ${}^{a}$	Yes ${}^{c}$
roboception	rc_visard	1280 × 960	3	1	Yes	Yes ${}^{a}$	No

^a^: functionality by open-source SDK; ^b^: functionality requires additional software; ^c^: limited to specific objects.

**Table 2:** Description of partial methods, data types used, and whether synchronization is required.

	Submodules	Image	Point Cloud	Synchronization
S1	Calibration	x		no
S2	Background Subtraction	x	x *	yes
S3	Merging and Voxelization		x	yes
S4	Segmentation	x **	x *	no
S5	Classification		x	no

*: only utility; **: projected image.

**Table 3:** Composition of validation scenarios with item classes and their appearance per scenario.

Item Class	1 Item	2 Items	3 Items	4 Items	SUM
Bar	2	2	2	0	6
Clamp	2	2	4	3	11
Silicone Gun	2	2	5	3	12
Hammer	2	3	5	6	16
Drill	2	4	4	3	13
Tongs	2	3	4	3	12
Rotary Disk	2	1	5	3	11
Rotary Table (LA)	2	1	3	3	9
Rotary Table (Full)	2	2	1	0	5
Total Scenarios	18	10	11	6	-

“LA” is the lower assembly; “full” is the full assembly (LA + rotary disk).

**Table 4:** Confusion matrix based on 9652 sampled items.

	Item Expected	No Item Expected
Item segmented	8407 (TP)	1245 (FN)
No item segmented	22 (FP)	0 (TN)

## Data Availability

SHAREWORK aims to publish data on Zenodo once the reports and project have been approved by the European Union. Partial data available on request.

## Abbreviations

The following abbreviations are used in this manuscript:

AR	Augmented Reality
CAD	Computer-Aided Design
CNN	Convolutional Neural Network
CPU	Central Processing Unit
DGCNN	Dynamic Graph Convolutional Neural Network
DIN	Deutsche Industrienorm
FLAME	Fuzzy Clustering by Local Approximation of Memberships
FN	False Negatives
FP	False Positives
GPU	Graphics Processing Unit
IGMR	Institute of Mechanism Theory, Machine Dynamics, and Robotics
kNN	*k*-Nearest Neighbors
LA	Lower Assembly
LiDAR	Light Detection and Ranging
MOG	Mixture of Gaussians
MP	Megapixel
n.a.	not available
openCV	Open Computer Vision
PCL	Point Cloud Library
RAM	Random Access Memory
RGB	Red Green Blue
ROI	Region of Interest
ROS	Robot Operating System
RSCNN	Relation-Shape Convolutional Neural Network
RWTH	Rheinisch Westphaelische Technische Hochschule
SDK	Software Development Kit
SHAREWORK	Safe and effective HumAn-Robot coopEration toWards a better cOmpetiveness
	on cuRrent automation lacK manufacturing processes
SIFT	Scale-Invariant Feature Transform
TN	True Negatives
TP	True Positives
